# MAIT Cells in the Bone Marrow of Patients with Aplastic Anemia

**DOI:** 10.3390/ijms251810160

**Published:** 2024-09-21

**Authors:** Lam Quang Vu, J. Luis Espinoza, Hoang Thao Giang Nguyen, Shohei Mizuno, Akiyoshi Takami

**Affiliations:** 1 Division of Hematology, Department of Internal Medicine, Aichi Medical University School of Medicine, Nagakute 480-1195, Japan; quanglamvu1991@gmail.com (L.Q.V.);; 2 Faculty of Health Sciences, Kanazawa University, Kanazawa 920-1192, Japan; luis@staff.kanazawa-u.ac.jp (J.L.E.); nhtgiang@stu.kanazawa-u.ac.jp (H.T.G.N.)

**Keywords:** mucosal-associated invariant T cells, aplastic anemia, bone marrow failure, autoimmune disorder, MAIT cells

## Abstract

Mucosal-associated invariant T cells (MAIT cells) are a subset of T cells with innate, effector-like properties that play an essential role in the immune response to microbial infections. In humans, MAIT cells are detectable in the blood, liver, and lungs, but little is known about the frequency of these cells in the bone marrow. Also, the pathogenic role, if any, of MAIT cells in the development of aplastic anemia, a disease with an exquisite origin in the bone marrow, is currently unknown. We investigated the frequency and clinical relevance of bone marrow MAIT cells in a cohort of 14 patients (60.6 ± 23 and 57% women) with aplastic anemia. MAIT cells in the bone marrow samples obtained at diagnosis were evaluated by flow cytometry, and their association with various blood cell parameters and the patients’ clinical features was analyzed. MAIT cells were detectable in the bone marrow of all patients, with considerable variations among them. Bone marrow MAIT cells expressing the activator receptor natural killer group 2D - NKG2D (NKG2D+ MAIT cells) were significantly more abundant in the specimens of the aplastic anemia patients than in patients with bone marrow failure distinct from aplastic anemia. In addition, the NKG2D+ MAIT cells positively correlated with whole blood cell counts (WBC), platelet counts, and neutrophil counts, as well as with various inflammatory markers, including neutrophil-to-lymphocyte rate (NLR), platelet-to-lymphocyte rate (PLR), and systemic inflammatory index (SII). In functional studies, bone marrow CD34+ hematopoietic cells exposed to phytohemagglutinin or bacterial-derived lipopolysaccharide and acetyl-6-formylpterin upregulated MR1 (major histocompatibility complex, class I-related, known to interact with MAIT cells) and MICA/B (MHC class I chain-related gene A, a ligand of NKG2D) proteins on their cell surface, suggesting that under stress conditions, CD34+ hematopoietic cells are more likely to interact with NKG2D+ MAIT cells. In addition, NKG2D+ MAIT cells upregulated perforin and granzyme B in response to their interaction with recombinant MICA protein *in vitro*. This study reports for the first time the frequency of MAIT cells in the bone marrow of patients with aplastic anemia and assesses the potential implications of these cells in the pathogenesis or progression of aplastic anemia.

## 1. Introduction

Aplastic anemia is a rare blood disorder characterized by pancytopenia and bone marrow failure (BMF) due to the loss of most hematopoietic stem and progenitor cells (HSPCs). This condition is often caused by an aberrant immune response, primarily mediated by cytotoxic T lymphocytes (CTLs), which recognize and destroy HSPCs [1,2]. Despite extensive research for decades, the specific molecular mechanisms underlying this autoimmune response, including the triggering factors and the specific antigens recognized by CTLs, have yet to be discovered. A plausible hypothesis proposes that a stress-inducible event, such as microbial infection, drug exposure, or specific environmental agents, may induce the immune response and, likely by molecular mimicry, cross-react with the antigens expressed on HSPCs [3,4]. The autoimmunity mechanism implicated in the origin of aplastic anemia is supported by the excellent response to immunosuppressive therapy (IST) with anti-thymocyte globulin (ATG) or cyclosporine observed in the vast majority of aplastic anemia patients, as well as the relatively higher frequency of the disease among individuals with specific geographic and genetic backgrounds and by the predilection of certain HLA risk alleles [5].

Mucosal-associated invariant T cells (MAIT cells) are a subpopulation of T cells that recognize riboflavin-derivative antigens from various microbial species presented by the major histocompatibility complex (MHC) class I-like protein MR1. As is the case for all T cell subsets, MAIT cells develop in the thymus; however, contrary to conventional T cells that undergo selection based on MHC class I or II molecules, MAIT cells are selected based on their interaction with MR1 [6]. MAIT cells display both innate and adaptive immune functions, having the ability to exert immune effector-like functions immediately after leaving the thymus, undergo clonal expansion in the peripheral tissues, and establish antigen memory. In healthy humans, MAIT cells constitute about 5% of the circulating T cell population and are detectable in various tissues, including the lungs, liver, joints, blood, and mucosal tissues [6,7]. Due to their interaction with molecules presented via MR1, MAIT cells play essential roles in the human immune response to bacterial and viral infections [7]. MAIT cells have been implicated in various inflammatory and autoimmune disorders, such as multiple sclerosis, inflammatory bowel disease, and chronic obstructive pulmonary disease [8]. Notably, even though aplastic anemia is an autoimmune disease with considerable gaps in the understanding of its pathogenic mechanism, especially regarding the autoantigens recognized by pathogenic T cells that elicit the autoimmune response in this disease, little is known about the role, if any, of MAIT cells in aplastic anemia. Importantly, MAIT cells play an essential role in the immune response to pathogenic bacteria, and the indirect evidence suggests that, at least in some patients, microbial infections may trigger the autoimmune response implicated in the development of bone marrow failure syndromes like aplastic anemia [9]. Here, we investigated the frequency of MAIT cells in bone marrow specimens collected at diagnosis in Japanese patients with confirmed aplastic anemia. The potential relevance of bone marrow MAIT cell numbers in clinical outcomes was also investigated. 

## 2. Results

The mean age of the patients included in this Japanese case series was 60 ± 23 years, with eight of them (52%) being females and six (43%) men. Among them, nine (64.3%) were found to have a normal karyotype, three (21.4%) had aneuploidy, and two (14.3%) presented structural abnormality after chromosomal analysis of the bone marrow aspirates. The patients were treated following the standard IST protocol with cyclosporine and supportive care. One patient required ATG and one patient also received steroids. Two patients received allogeneic hematopoietic cell transplantation and one patient progressed to myelodysplastic syndrome (MDS) (Table 1).

**Figure 1:**
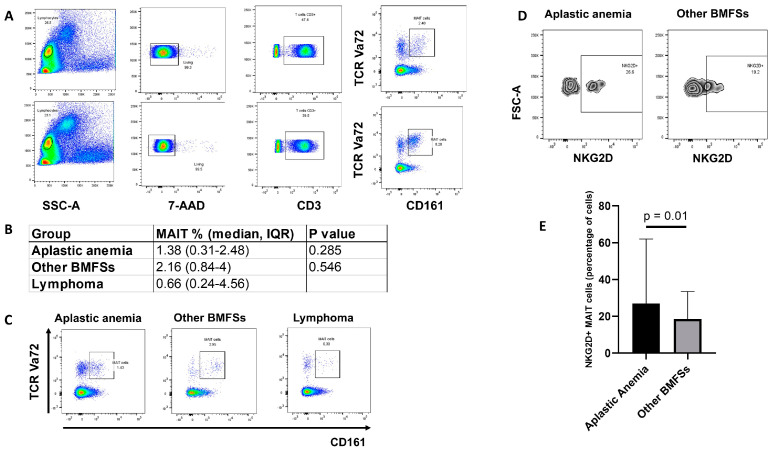
(**A**) The flow cytometry gating strategy to identify and enumerate the mucosal-associated invariant T (MAIT) cells in the bone marrow of the patients included in this study. The upper panel depicts actual data from a patient with low MAIT cell numbers, and the bottom panel shows data from a patient with high MAIT cell numbers. (**B**) The chart summarizes the MAIT cell percentages in the bone marrow of patients with aplastic anemia in comparison with those with lymphoma, or with other bone marrow failure syndromes (BMFSs). The numbers represent the median IQRs. (**C**) Representative flow cytometry dot plots showing the frequency of MAIT cells in the bone marrow of patients with aplastic anemia, lymphoma, and other bone marrow failure syndromes (BMFSs). (**D**) Representative flow cytometry dot plots from a patient with aplastic anemia and a patient with a different bone marrow failure syndrome showing the frequency of MAIT cells expressing the activator receptor NKG2D. (**E**) The graph summarizes the percentages of MAIT cells expressing the activator receptor NKG2D (NKG2D+ MAIT cells) in the bone marrow of the patients with aplastic anemia in comparison with that of the patients with other cytopenias distinct from aplastic anemia (BMFSs). The numbers represent the median IQRs. TCR Va72: T-cell antigen receptor V-alpha7.2; NKG2D: natural killer group 2D.

MAIT cells, categorized as CD3+, CD161+, and TCR Va72+ (T-cell antigen receptor V-alpha7.2) lymphocytes, were detectable in the bone marrow of all patients, with considerable variations among them (1.38 (0.31–2.48) median, IQR) (Figure 1A). Of note, the bone marrow MAIT cells were above the median (4.07%) in the patient that required ATG. This patient also required hematopoietic cell transplantation, while in the patient that required steroids, the MAIT cells were 1.91%,. Since there are no previous publications reporting the percentages of MAIT cells in the bone marrow of healthy individuals, bone marrow specimens from healthy individuals are not routinely obtained due to ethical issues and the invasive nature of the procedure; we therefore enumerated the MAIT cells in the bone marrow of 10 patients with diffuse large B cell lymphoma (DLBCL) without bone marrow involvement, abnormal blood cell counts, or extranodal disease (Table 2).

Although these patients do not represent truly “normal” bone marrow, we used the number of MAIT cells from these DLBCL patients as a reference to estimate the MAIT cell population in the bone marrow in the absence of malignancy or bone marrow failure. In the lymphoma patients, the median IQR of the bone marrow MAIT cells was 0.66 (0.24–4.56), which is slightly lower than that observed in aplastic anemia, although these differences were not statistically significant (*p* = 0.28).

Interestingly, the median IQR of the bone marrow MAIT cells in the aplastic anemia cohort was slightly lower than that observed in the cohort of 10 patients who were investigated in our institution in the same period of time for bone marrow failure, but were confirmed to not have aplastic anemia. This included four patients with MDS, four patients with isolated thrombocytopenia, and two patients with drug-induced aplastic anemia (Table 3).

In this group, the median IQR of the bone marrow MAIT cells was 2.16 (0.84–4.0), although it did not reach statistical significance (*p* = 0.54) (Figure 1B).

To investigate the biological and clinical relevance of the bone marrow MAIT cells in patients with aplastic anemia, we analyzed the potential correlations between MAIT cell numbers and various parameters derived from whole blood cell counts (WBC) and chemical blood tests, many of which have been reported to be associated with autoimmune and inflammatory disorders [10,11,12]. We observed that MAIT cell numbers were inversely correlated with RDW (r2 = −0.639, *p* = 0.014). Other factors, including total leukocyte counts and whole blood cell differential, as well as blood chemical markers, were not associated with MAIT cell counts (Table 4).

Notably, the percentage of MAIT cells expressing the activator receptor NKG2D was significantly higher in the bone marrow specimens of the aplastic anemia patients than in MAIT cells from the patients with other bone marrow syndromes [median, IQR 26.5% (22.44–50.5) vs. 17.9% (6.25–21), *p* = 0.01] (Figure 1E). Thus, considering the important relevance of the NKG2D receptor in various human immune response processes, including autoimmunity and the immune response to infections, we analyzed the potential association of the NKG2D+ MAIT cells with various parameters derived from WBC. Our analysis revealed that the percentage of NKG2D+ MAIT cells positively correlated with platelet count, WBC, neutrophil count, NLR, PLR, and SII (Table 5), suggesting the possibility that NKG2D+ MAIT cells could be implicated in the inflammatory response associated with aplastic anemia.

**Figure 2:**
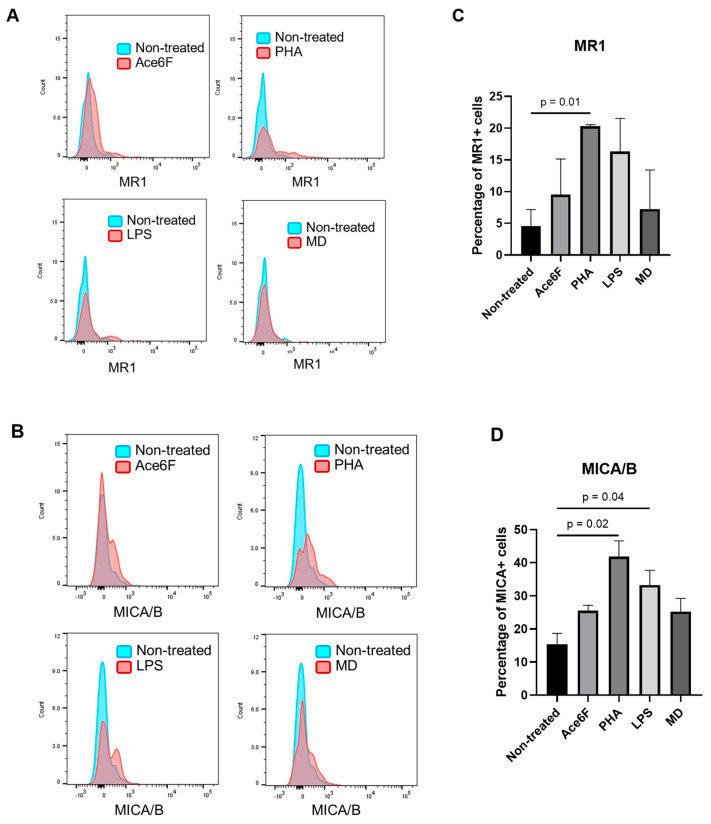
(**A,B**) MR1 and MICA/B expression in hematopoietic stem cells. The representative histograms showing the expression of MR1 (**A**) and MICA/b (**B**) in CD34+ bone marrow hematopoietic cells cultured for 48 h in the presence or the absence of flow cytometry analysis demonstrate the expression of MR1 and MICA/B between non-treated and treated with different inflammatory agents in the bone marrow samples from the lymphoma patients after 48 h. The summarized data of bone marrow cells derived from three different lymphoma patients comparing the percentage of MR1 positive cells (**C**) and MICA/B positive cells (**D**) after treating 48 h of culture. MR1: major histocompatibility complex, class I-related; MICA/B: MHC class I chain-related gene A /B; Ace6F: acetyl-6-formylpterin; PHA: phytohemagglutinin; LPS: lipopolysaccharide; MD: muramyl dipeptide.

We next investigated the expression ofmajor histocompatibility complex, class I-related (MR1, a protein that that directly interacts with MAIT cells) and MHC class I chain-related gene A/B (MICA/B, a ligand for the activator receptor NKG2D) in CD34+ bone marrow cells in the presence or absence of bioactive or microbe-derived factors. We utilized CD34+ hematopoietic cells from the bone marrow of the lymphoma patients, since the bone marrow of aplastic anemia patients is known to contain very few CD34+ hematopoietic cells. Our analysis revealed that all inflammatory factors stimulated the expression of both MR1 and MICA/B on the surface of the CD34+ cells. We observed that whereas MR1 and MICA/B proteins were almost absent on the surface of non-treated cells, in cells exposed to PHA, both MR1 (*p* = 0.01) and MICA/B (*p* = 0.02) were significantly upregulated. Similarly, LPS increased the expression of both proteins (*p* = 0.04). Additionally, Ace6F, which was previously reported to increase MR1 on various cell lines [13], certainly augmented the percentage of MR1-expressing CD34+ cells and also enhanced the number of MICA/B+ cells to around 25%. On the other hand, although the microbe-derived factor MD induced a mild increase in the expression levels of the examined markers, these effects were not statistically significant (Figure 2).

**Figure 3:**
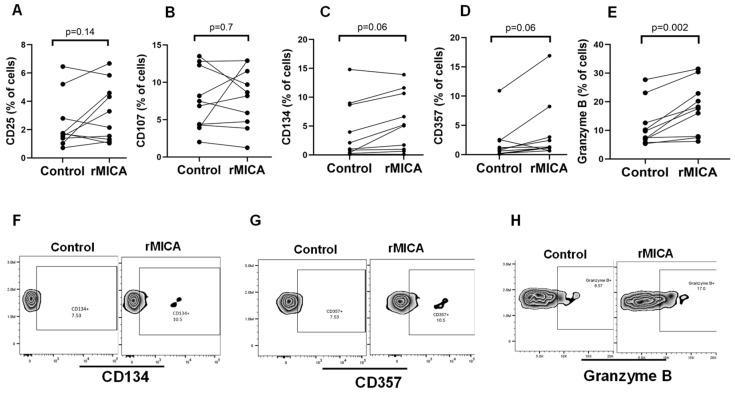
The in vitro activation of bone marrow mucosal-associated invariant T (MAIT) cells after their interaction with recombinant MHC class I chain-related gene A (rMICA). The bone marrow mononuclear cells were cultured in the presence or absence of plate-coated rMICA for 24 h and thereafter stained with MAIT cell antibodies and antibodies specific to the markers of T cell inflammation, including CD25 (**A**), CD107 (**B**), CD134 (**C**), CD357 (**D**), and granzyme B (**E**). The figures show the percentage of cells expressing the indicated marker, with each dot indicating the individual data from the control cells or the cells exposed to rMICA. Representative flow cytometry images that depict the expression of CD134 (**F**), CD357 (**G**), and granzyme B (**H**) are also shown.

Finally, to substantiate the relevance of NKG2D receptor expression on bone marrow MAIT cells, we cultured bone marrow MAIT cells derived from 10 patients with aplastic anemia in the presence or absence of recombinant MICA (rMICA), one of the NKG2D ligands (NKG2D-Ls), and assessed the potential changes in the expression of various cell surface proteins known to be upregulated in activated T cells. We observed considerable interindividual variations in the expression of markers of T cell activation, and while the expression of CD25 (Figure 3A) and CD107 (Figure 3B) did not change significantly after NKG2D receptor interaction with rMICA, the expression of CD134 (Figure 3C,F), CD357 (Figure 3D,G), and Granzyme B (Figure 3E,H) increased in MAIT cells from most of the patients tested, although these changes were statistically significant only for granzyme B.

**Figure 4:**
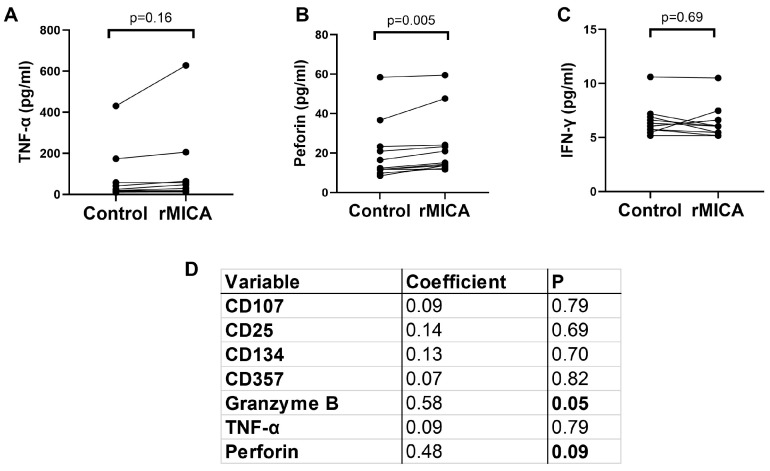
The supernatants of the cells cultured as described in Figure 3 were subjected to ELISA analysis for the detection of TNF-α (**A**), IFN-γ (**B**), and perforin A (**C**). The figures show the concentrations of the indicated analytes, with each dot indicating the individual data from the control cells or the cells exposed to recombinant MHC class I chain-related gene A (rMICA). (**D**) An analysis of the correlation between the baseline expression of the NKG2D receptor on the bone marrow mucosal-associated invariant T (MAIT) cells and the fold change of the indicated analytes (cell surface markers and cytokines) after the cell exposure to rMICA for 24 h.

An analysis of the cell culture supernatants showed significantly higher levels of secreted perforin in the cells exposed to rMICA, while tumor necrosis factor alpha (TNF-α) did not change significantly and interferon gamma (IFN-γ) was almost undetectable in the supernatants (Figure 4A). Interestingly, the changes in the expression of cell activator makers and secreted cytokines showed a positive correlation with the NKG2D expression in the bone marrow MAIT cells before stimulation, this correlation being more evident for granzyme B and perforin (Figure 4B).

Collectively, these results suggest that NKG2D expression on the surface of bone marrow MAIT cells of patients with aplastic anemia may elicit specific changes in the bone marrow environment after its interaction with surrounding cells expressing NKG2D-Ls.

## 3. Discussion

Aplastic anemia is a very rare blood disorder that, in the majority of cases, is caused by an autoimmune response triggered by a yet unknown mechanism. Several studies have shown that pathogenic cytotoxic T cells are involved in the autoimmune attack implicated in the destruction of the hematopoietic stem cells in the bone marrow. This study reports for the first time the enumeration and analysis of MAIT cells in bone marrow specimens of patients with newly diagnosed aplastic anemia. Our data reveals that MAIT cells were detected in all bone marrow specimens, with variable numbers among the 14 aplastic anemia patients studied. Importantly, our data revealed that bone marrow NKG2D+ MAIT cells were significantly more abundant in the specimens of the aplastic anemia patients than those observed among patients with cytopenias without aplastic anemia. In addition, the NKG2D+ MAIT cells positively correlated with various inflammatory markers, including WBC, NLR, PLR, SII, platelet counts, and neutrophil counts.

In healthy humans, MAIT cells constitute up to 5% of the peripheral T cell population, while they are highly abundant in the liver (20–40% of the T cells) and are also detectable in the lungs, liver, joints, blood, and intestinal mucosa. Despite the importance of MAIT cells in the immune response to infections and their implications for autoimmune diseases, little is known about the frequency of these cells in patients with hematological diseases. One study reported that MAIT cells in the peripheral blood of patients with newly diagnosed multiple myeloma were significantly lower (mean 0.83%, range 0.02–2.8%) than those in their healthy control counterparts (mean 3.04%, range 0.07–17.6%) [14]. More recently, Peng et al. [15] analyzed MAIT cells in peripheral blood or bone marrow samples of patients with acute myeloid leukemia (AML). Compared to healthy controls, the newly diagnosed AML patients exhibited a significant decrease in the frequency of MAIT cells in the peripheral blood (mean: 3.12% vs. 6.19%). Interestingly, a paired analysis comparing bone marrow sample aspirates with matched peripheral blood samples from the newly diagnosed AML patients showed that the frequency of MAIT cells in the bone marrow almost mirrored that in the peripheral blood (MAIT cell mean: 3.08% vs. 3.01%), suggesting a direct correlation of the peripheral blood MAIT cells with those in the bone marrow microenvironments of the patients with AML [15].

The role of MAIT cells in the pathogenesis of aplastic anemia, whether promoter or protective cells, is unclear. To the best of our knowledge, this is the first study to investigate the frequency or potential significance of MAIT cells in the bone marrow of patients with aplastic anemia. Whether NKG2D+ MAIT cells are directly implicated in the pathogenesis of immune-mediated aplastic anemia is currently unknown. Previous studies have reported the expression of the NKG2D receptor in circulating MAIT cells [16], although the biological significance of this receptor has not been functionally investigated; based on what we know from other immune cells, including NK cells and CD8+ T cells, one may expect that the MAIT cells expressing the NKG2D receptor are capable of recognizing target cells expressing NKG2D-Ls, including MICA/B and ULBPs. Importantly, previous studies have reported that hematopoietic progenitor cells can express NKG2D-Ls when exposed to stress signals [17,18] becoming potentially susceptible to an immune attack elicited by NKG2D receptor-expressing immune cells. Similarly, in in vitro studies, immature myeloid cells have been shown to upregulate MR1 proteins (the targets of MAIT cells) on their surface when exposed to inflammatory stimuli [13]. Considering that HSPCscan be directly infected by various microorganisms [9,19], it is plausible that infection with certain pathogens could induce the expression of MR1 and/or NKG2D-Ls on the surface ofHSPCs, potentially triggering an autoimmune attack mediated by MAIT cells. Our findings showing the upregulation of MICA/B and MR1 on the cell surface of CD34+ bone marrow cells when exposed to PHA and LPS, and to a lesser extent Ace6F, are consistent with this notion; however, given the technical limitations associated with the difficulty in obtaining HSPCs from patients with aplastic anemia due to the bone marrow destruction inherent to the disease, we were not able to prove experimentally this hypothesis. Utilizing in vitro-expanded HSPCs could be a potential technical solution for these limitations, since HSPCs from patients with aplastic anemia can be efficiently generated in vitro utilizing iPS cell technology [3]. Unfortunately, aplastic anemia-derived iPS cells were not available for the current study, but we believe this hypothesis is worth testing in the future. Nevertheless, our findings that after their in vitro exposure to rMICA, bone marrow NKG2D+ MAIT cells showed cell activation features including the upregulation of granzyme B, perforin, and to some extent CD134 and CD357, provide indirect evidence of the functional relevance of the NKG2D receptor in those cells and suggest a potential implication of the NKG2D axis in the pathogenesis of this disease. Importantly, granzyme B and perforin constitute the main components of the cytotoxic cargo of cytotoxic lymphocytes (cytotoxic T lymphocytes and NK cells) and thereby mediate target cell death [20]; thus, the upregulation of granzyme B and perforin in the bone marrow NKG2D+ MAIT cells after their exposure to the NKG2D-Ls is consistent with the reported ability of these cells to recognize viral infections [21]. However, whether bone marrow NKG2D+ MAIT cells can recognize and eliminate HSPCs is currently unknown.

The MR1 protein is capable of binding to molecules derived from bacterial riboflavin biosynthesis and then presenting them to the MAIT cells for activation, and several human cell types are capable of presenting antigens via MR1 with varying efficiency. As mentioned above, one of the most accepted putative mechanisms underlying the pathogenesis of aplastic anemia proposes that a microbial infection triggers the emergence of pathogenic CTLs, which eventually target HSPCs in the bone marrow by antigenic molecular mimicry. This is supported by the documented cases of the emergence of aplastic anemia following infection with a range of pathogens [9]. In this regard, one may speculate that microbial infection involving HSPC or the surrounding cells could induce the expression of MR1 on HSPCs, which in turn may present microbe peptides to the MAIT cells via MR1, thereby leading to HSPC killing. However, it cannot be ruled out that MAIT cells may have a protective role for HSPCs in aplastic anemia, as preclinical studies using experimental models of lung metastasis showed that MAIT cells promoted tumor initiation, growth, and metastasis by suppressing T and/or NK cells [22].

Our analysis showed that the aplastic anemia patients with higher percentages of NKG2D+ MAIT cells had elevated platelet counts, raising questions about how MAIT cells might influence platelet production and maturation. One potential mechanism involves the immunomodulatory functions of MAIT cells, which produce cytokines like IL-17 and IFN-γ that regulate thrombopoiesis [7]. IL-17 has been shown to stimulate thrombopoietin (TPO) production, which is crucial for promoting megakaryocyte maturation and increasing platelet counts [23,24]. TPO, alone or in combination with other cytokines, accelerates megakaryocyte differentiation and enhances platelet production. Additionally, IL-17 can enhance the interaction with its receptor, regulating processes crucial for megakaryocyte development and platelet formation [25]. These findings support the hypothesis that increased IL-17 levels in patients with a higher percentage of MAIT cells could lead to elevated TPO production, promoting megakaryocyte maturation and resulting in higher platelet counts and a greater proportion of immature platelets in the bloodstream. Furthermore, studies on inflammatory diseases have shown that activated MAIT cells exhibit increased NKG2D expression and cytokine production [26,27], suggesting that their activation status enhances their functional capabilities. This supports our findings, indicating that in aplastic anemia, the higher percentage of NKG2D+ MAIT cells might contribute to a more favorable bone marrow environment for platelet production through their enhanced cytokine production and immune regulatory functions. The increase in NKG2D+ MAIT cells in aplastic anemia may arise as a compensatory response to thrombocytopenia in the early stages of the disease. This suggests that, in the future, developing therapies to modulate NKG2D+ MAIT cell numbers could potentially aid hematopoietic recovery in aplastic anemia patients. However, this remains highly speculative, and further research is needed to validate this potential therapeutic approach.

MAIT cells may influence the bone marrow microenvironment by modulating the immune response and interacting with other cells, such as stromal cells and HSPCs. Their ability to produce cytokines like IL-17 and IFN-γ could affect the proliferation and differentiation of HSPCs, potentially impacting hematopoiesis. Additionally, the presence of MAIT cells might alter the inflammatory milieu of the bone marrow, influencing the behavior of both healthy and malignant cells. MAIT cells are also known for their role in immune surveillance, particularly in recognizing microbial metabolites presented by MR1. Their rapid response to infection and ability to produce a range of cytokines, including IL-17 and IFN-γ, position them as crucial players in the bone marrow’s immune environment. Interactions between MAIT cells and other immune cells, such as NK cells and macrophages, are pivotal in shaping the immune response within the bone marrow. Understanding these interactions could provide insights into the broader immune regulatory networks in aplastic anemia.

There are various limitations associated with this study. First, the small sample size limits the generalizability of our findings. Second, due to the unavailability of healthy bone marrow, we used bone marrow from DLBCL patients confirmed to be free of malignant cell infiltration as controls, which may not perfectly represent normal conditions. Third, we could not analyze the MAIT cell percentages in the peripheral blood, as peripheral blood mononuclear cells collected at diagnosis were unavailable for all patients included in the study. Fourth, only bone marrow samples collected at the time of diagnosis were available for this study, preventing us from assessing the potential changes in MAIT cell percentages during treatment and follow-up. These limitations highlight the need for further studies with larger sample sizes, including healthy controls and longitudinal monitoring, to fully understand the role of MAIT cells in aplastic anemia. Given the rarity of the disease and the fact that this is a single-center study, we were able to study a relatively small number of cases, which may have affected the statistical power of the study and, therefore, limited our ability to draw definitive conclusions. 

## 4. Materials and Methods

### 4.1. Patients and Study Design

Patients being investigated for bone marrow failure (BMF), including 14 patients with confirmed immune-mediated aplastic anemia and 10 patients with other BMFSs, and treated at Aichi Medical University Hospital were enrolled in this study. Bone marrow specimens from these patients were collected between September 2017 and February 2022. The diagnosis of aplastic anemia was performed following the national guidelines for the diagnosis and treatment of idiopathic disorders, as accessed on 5 January 2022. (http://zoketsushogaihan.umin.jp/file/2022/Aplastic_Anemia.pdf).

Bone marrow specimens from ten patients with diffuse large B cell lymphoma (DLBCL), collected between March 2020 and March 2022 and confirmed to be free of bone marrow infiltration from malignant cells, were also utilized for this study. The diagnosis of DLBCL was made according to the recently updated diagnosis guidelines [28]. Relevant data, including clinical history, hematological examination findings, and treatments, were retrospectively collected from electronic medical records. The study was conducted according to the principles of the Declaration of Helsinki of 1975, as revised in 2013, and ethical approval was obtained from the Institutional Review Board of Aichi Medical University School of Medicine.

### 4.2. Collection and Storage of Bone Marrow Samples

The bone marrow specimens were collected from newly diagnosed patients at the department of hematology at Aichi Medical University Hospital. Bone marrow mononuclear cells were isolated from the bone marrow samples using the Ficoll gradient method, following the manufacturer’s instructions (Sigma-Aldrich Ficoll^®^-Paque PREMIUM 1.073, Sigma-Aldrich, St. Louis, MO, USA). The purified cells were then resuspended in Cellbanker freezing medium (Nippon Zenyaku Kogyo, Koriyama, Fukushima, Japan) and stored at −80 °C until use.

### 4.3. Blood Cell Counts, Blood Chemistry, and Blood Cell Rate Calculations

Blood chemistry and blood cell counts and differentials were measured at the central clinical laboratory of Aichi Medical University using an XN-9000 (Sysmex Corporation, Kobe, Hyogo, Japan) for blood cell counting and a LABOSPECT008 (Hitachi-Technologies Corporation, Tokyo, Japan) with a colorimetric assay for the blood chemistry. The neutrophil-to-lymphocyte ratio (NLR) and platelet-to-lymphocyte ratio (PLR) were calculated by dividing the neutrophil and platelet counts by the lymphocyte count, respectively. The systemic inflammatory index (SII) was calculated by dividing the products of the neutrophil counts and platelet counts by the lymphocyte counts and expressed as G/L.

### 4.4. Induction of MR1 and MICA Expression on Hematopoietic Stem Cells

The bone marrow cells from three lymphoma patients were cultured in RPMI-1640 (#189-02025; Wako-Fujifilm, Tokyo, Japan) supplemented with 20% of fetal bovine serum (S-FBS-NL-015; Serana, Brandenburg, Germany). The following growth factors (working concentration: 10 ng/mL each) were added to the culture medium: human stem cell factor (#8925; Cell Signaling Technology, Danvers, MA, USA); granulocyte colony-stimulating factor (#CSB-AP002081HU; WUHAN HUAMEI BIOTECH Co., Ltd.;, Wuhan, China); and human Flt-3 Ligand/FLT3L (#308-FKHB; R&D Systems, Minneapolis, MN, USA) at 37 °C and 5% CO_2_. The cultured cells were treated with the following bioactive or microbe-derived factors: Acetyl-6-formylpterin (Ace-6F, #23303; Cayman, Ann Arbor, MI, USA); Phytohemagglutinin (PHA, #L8902; Sigma-Aldrich, St. Louis, MO, USA); Lipopolysaccharide (LPS, #19660, Cayman, Ann Arbor, MI, USA); and Muramyl dipeptide (MD, #30866; Cayman, Ann Arbor, MI, USA). After 48 h of culture, the cells were analyzed by flow cytometry.

### 4.5. Assessing Immune Response via NKG2D/MICA Interaction on MAIT Cells

Recombinant MICA/Fc chimera (rMICA), a ligand of NKG2D, was purchased from R&D Systems (#1300-MA-050) and diluted to a concentration of 1 µg/mL in sterile PBS, and 1 mL of the solution was added to each well of a 24-well plate. The plate was sealed and incubated overnight at 4 °C. The following day, the solution was aspirated, and the plate was washed three times with PBS before use. The bone marrow cells from ten aplastic anemia patients were quickly thawed and cultured in RPMI-1640 (#189-02025; Wako-Fujifilm, Tokyo, Japan) with 20% of fetal bovine serum (FBS) and supplemented with IL-2 (~10 IU/mL). Each bone marrow sample was divided into two parts: one part was cultured on a MICA-coated plate, while the other was cultured on a non-coated plate. All samples were incubated at 37 °C with 5% CO_2_. After 24 h, the supernatants were collected for cytokine concentration analysis, while the cells were stained with conjugated antibodies for flow cytometry.

### 4.6. Flow Cytometry and ELISA

The cryopreserved bone marrow samples were quickly thawed in a culture medium (RPMI supplemented with 10% FBS), warmed at 37 °C, and then stained with the following antibodies at 4 °C for 20 min: CD3-FITC (#300406); CD161-APC (#339912); TCR Vα7.2-Brilliant Violet 421 (#351716); NKG2D APC-C7 (#320823); and 7-AAD Viability Staining Solution (#420403) to exclude dead cells. All these antibodies and reagents were purchased from BioLegend (San Diego, CA, USA). In the other experiments, the flow cytometry studies were performed on bone marrow cells from the patients after 24 or 48 h of culture and involved staining with the following antibodies: anti-CD34-FITC (#34360), anti-MR1-PE (#361105), anti-MICA/B-APC (#320907), anti-CD107a-PECy7 (#328617), anti-CD25 (#302611), and anti-CD357 (#371224) from BioLegend, as well as anti-Granzyme-B-PE (#561142), anti-CD278-PE (#557802), anti-CD314 (#557940), and anti-CD134 (#555838) from BD Pharmingen (Franklin Lakes, NJ, USA). The cells were analyzed using an LSR-Fortessa X-20 instrument (BD Biosciences, San Jose, CA, USA) and NovoSampler Pro (Agilent, Santa Clara, CA, USA). Data were analyzed using FlowJo software (ver. 10; Tree Star, Ashland, OR, USA). The cell supernatants collected as described in Section 4.5 were assessed by an enzyme-linked immunosorbent assay (ELISA), utilizing commercially available specific assay kits for measuring the concentration of TNF-α (#3512-2H), IFN-γ (#3420-1H-6), and perforin A (#3465-2H), all from Mabtech (Nacka Strand, Sweden).

### 4.7. Statistical Analysis

Spearman’s rank correlation coefficient was utilized for the correlation analysis. Continuous variables were presented as medians with 25–75% interquartile ranges (IQRs). A Mann–Whitney U test was used to compare medians between the two groups. Categorical variables were shown as percentages (%) and compared using the chi-square test or Fisher’s exact test. Changes in the expression of surface markers on the MAIT cells or in the levels of cytokines in their culture supernatants after their in vitro exposure to rMICA were assessed by a paired t-test. The fold changes of the cytokines or cell surface markers were calculated as the ratio of the difference between the final value (value after rMICA treatment) and the initial value (baseline value) over the initial value. Namely, if the baseline value was A and the final value was B, the fold change was (B − A)/A. A *p* value of less than 0.05 was considered to indicate statistical significance. The statistical analyses were carried out with GraphPad Prism 8 (San Diego, CA, USA) and the SPSS software package version 25.0 (IBM Corp., Armonk, NY, USA).

## 5. Conclusions

This study reports for the first time the enumeration and analysis of the clinical relevance of MAIT cells in the bone marrow of patients with aplastic anemia. Considerable variations in MAIT cell numbers were observed among the patients included in the study. Further research is required to explore these mechanisms and to confirm these findings in larger cohorts, including healthy controls and longitudinal studies.

## Figures and Tables

**Table 1:** The clinical characteristics of the aplastic anemia cohort.

Patient	Age	Gender	MAIT (%)	MDS Conversion	AHCT	Chromosome Analysis
AA1	82	F	0.19	No	No	46, XX 20/20
AA2	83	M	0.17	No	N/A	46, XY 20/20
AA3	74	M	0.33	No	No	46, XY 20/20
AA8	84	M	15.0	No	No	46, XY, t(4;4)(p16;q31) 1/20 46, XY 19/20
AA9	52	F	6.27	No	N/A	46, XX, t(10;15)(q26;q24) 1/20 46,XX 19/21
AA10	40	F	0.98	No	No	46, XX 20/20
AA11	69	M	1.13	No	No	46, XY 20/20
AA14	39	F	2.11	No	No	46, XX 20/20
AA16	56	M	0.97	Yes	Yes	46, XY 5/5
AA17	77	F	1.68	N/A	No	46, XY 20/20
AA20	68	F	1.18	No	No	47, XX, +8 4/20 46, XX 16/20
AA22	68	F	0.09	No	No	−20, +mar 1/20 46, XX 19/20
AA23	74	F	0.12	No	No	45, X, −X 11/11
AA27	18	M	1.39	No	Yes	46, XY 16/16

MDS: myelodysplastic syndrome; AHCT: allogeneic hematopoietic cell transplantation; MAIT: mucosal-associated invariant T cells.

**Table 2:** Key demographic and clinical characteristics of lymphoma cohort.

Patient	Type of Lymphoma	Age	Gender	MAIT Cell (%)
ML1	DLBCL (ND)	71	M	0.26
ML2	DLBCL (ND)	24	M	4.56
ML3	DLBCL (ND)	61	M	1.97
ML4	DLBCL (CR)	72	M	5.32
ML5	DLBCL (ND)	77	M	0.19
ML6	DLBCL (ND)	63	M	1.02
ML7	DLBCL (relapse)	83	F	0.20
ML8	DLBCL (ND)	89	M	0.24
ML9	DLBCL (ND)	82	F	0.30
ML10	DLBCL (relapse)	62	M	5.67

DLBCL: diffuse large B cell lymphoma; ND: newly diagnosed; MAIT: mucosal-associated invariant T.

**Table 3:** The key demographic and clinical characteristics of the other BMFS cohort.

Patient	Diagnosis	Age	Gender	MAIT (%)
BMFS 1	MDS	75	M	4
BMFS 2	Drug-induced aplastic anemia	69	M	0.8
BMFS 3	MDS (5q syndrome)	80	F	0.84
BMFS 4	MDS	81	M	2.05
BMFS 5	MDS	56	M	2.26
BMFS 6	Immune thrombocytopenia	75	F	1.95
BMFS 7	Thrombocytopenia	58	F	8
BMFS 8	Drug-induced aplastic anemia	68	F	0.097
BMFS 9	Immune thrombocytopenia	26	F	6.18
BMFS 10	Thrombocytopenia	74	M	2.95

BMFS: bone marrow failure syndrome; MDS: myelodysplastic syndrome, MAIT: mucosal-associated invariant T.

**Table 4:** The correlation between the percentage of bone marrow MAIT cells and various blood parameters.

Parameters	Coefficient	*p* Value
Age	−0.172	0.557
Hemoglobin	0.108	0.714
Hematocrit	0.062	0.834
RBC	0.158	0.589
RDW	−0.639	0.014
Reticulocytes	−0.328	0.253
Platelets	0.162	0.580
PDW	0.128	0.709
IPF	0.428	0.165
Total WBC	0.123	0.674
LDH	−0.059	0.840
Total serum protein	0.131	0.654
Vitamin B12	0.127	0.709
Folic acid	−0.122	0.738
NLR	0.216	0.459
PLR	0.216	0.459
SII	0.229	0.431

IPF: Immature platelet fraction; LDH: lactate dehydrogenase; PLR: platelet-to-lymphocyte rate; NLR: neutrophil–to-lymphocyte rate; RDW: red cell distribution width, SII: systemic inflammatory index; PDW: platelet distribution width; WBC: white blood cell count; RBC: red blood cell count. Correlations were determined by Spearman’s rank correlation coefficient.

**Table 5:** The correlation between the percentage of bone marrow NKG2D+ MAIT cells and various blood parameters.

Parameters	Coefficient	*p* Value
Age	0.432	0.123
MAIT%	0.194	0.507
Hemoglobin	−0.337	0.239
Hematocrit	−0.334	0.243
RBC	−0.378	0.182
RDW	0.117	0.690
Reticulocytes	0.042	0.887
Platelets	0.802	0.001
PDW	0.528	0.095
IPF	0.049	0.879
Total WBC	0.711	0.004
LDH (IFCC)	0.042	0.887
Total serumprotein	0.054	0.854
B12	−0.236	0.484
Folic acid	0.097	0.789
NLR	0.858	<0.001
PLR	0.748	0.002
SII	0.845	<0.001

IPF: Immature platelet fraction; LDH: lactate dehydrogenase; PLR: platelet–to-lymphocyte rate; NLR: neutrophil–to-lymphocyte rate; RDW: red cell distribution width; SII: systemic inflammatory index; PDW: platelet distribution width; WBC: white blood cell count; RBC: red blood cell count. Correlations were determined by Spearman’s rank correlation coefficient.

## Data Availability

The data associated with this study can be obtained from the corresponding author upon reasonable request.
